# Formation of Polychlorinated Biphenyls on Secondary Copper Production Fly Ash: Mechanistic Aspects and Correlation to Other Persistent Organic Pollutants

**DOI:** 10.1038/srep13903

**Published:** 2015-09-16

**Authors:** Xiaoxu Jiang, Guorui Liu, Mei Wang, Minghui Zheng

**Affiliations:** 1 State Key Laboratory of Environmental Chemistry and Ecotoxicology, Research Center for Eco-Environmental Sciences, Chinese Academy of Sciences, P.O. Box 2871, Beijing 100085, China

## Abstract

Emission of unintentionally formed polychlorinated biphenyls (PCBs) from industrial thermal processes is a global issue. Because the production and use of technical PCB mixtures has been banned, industrial thermal processes have become increasingly important sources of PCBs. Among these processes, secondary copper smelting is an important PCB source in China. In the present study, the potential for fly ash-mediated formation of PCBs in the secondary copper industry, and the mechanisms involved, were studied in laboratory thermochemical experiments. The total PCB concentrations were 37–70 times higher than the initial concentrations. Thermochemical reactions on the fly ash amplified the potential toxic equivalents of PCBs. The formation of PCBs over time and the effect of temperature were investigated. Based on analyses of PCB homologue profiles with different reaction conditions, a chlorination mechanism was proposed for forming PCBs in addition to a *de novo* synthesis mechanism. The chlorination pathway was supported by close correlations between each pair of adjacent homologue groups. Formation of PCBs and multiple persistent organic pollutants, including polychlorinated dibenzo-*p*-dioxins, polychlorinated dibenzofurans and polychlorinated naphthalenes, occurred during the tests, indicating that these compounds may share similar formation mechanisms.

Polychlorinated biphenyls (PCBs) are chlorinated aromatic compounds that are toxic, semi-volatile, stable and persistent in the environment. They are included in the Stockholm Convention on Persistent Organic Pollutants (POPs), the aim of which is to eliminate emissions of POPs globally. In the 1940s it was discovered that PCBs have adverse effects on biota, and this has led to concern about human and ecosystems exposure to trace concentrations of PCBs^1^,^2^. PCBs were detected in eagle tissue in 1944, and regional patterns of PCB contamination in eagles around the world were found to account for geographical variations in eggshell thickness^1^,^2^. Numerous studies since then have indicated that PCBs may contribute to severe health problems, such as cancer, immune suppression, and genotoxic effects^3^,^4^,^5^,^6^. Both prenatal and postnatal exposure to PCBs are negatively associated with cognitive functions during infancy and childhood^7^. The PCBs include 12 congeners that have been assigned toxicity equivalency factors by the World Health Organization because they exhibit dioxin-like toxicity. These congeners were classified as carcinogenic to humans (Group 1) by the International Agency for Research on Cancer in 2014.

Most PCBs in the environment are from technical PCB mixtures or are unintentionally formed in industrial processes. Until the end of the 1980s, PCBs were produced intentionally and used in many applications, including as heat-exchange fluids and additives because of their chemical stabilities. To date, approximately 1.3 million tonnes (1300 kg) of PCB have been produced globally, and still there are more than 9 million tonnes (9000 kg) of PCB containing oils and equipment that require remediation or active waste management to avoid further pollution of the environment^8^. Unintentionally produced PCBs have been detected in the environment, including in the Delaware river (USA), which was contaminated by a TiO_2_ plant that used chlorination technology^9^,^10^. Major sources of unintentionally produced PCBs include thermal industrial processes and production of technical chemicals. Unintentionally produced PCBs are found as by-products in some commercial chemicals, including pigments^11^,^12^,^13^, chloranil^14^ and 2,4-dichlorophenoxyacetic acid^15^. PCBs are also often produced as by-products of industrial activities, biomass and fossil fuel combustion, and other combustion processes. Because of unintentionally production of PCBs, public interest in control of PCB emissions has increased. In particular, thermal industries, such as ferrous and nonferrous metal smelting, thermal wire reclamation, and waste incineration, have come under scrutiny^16^,^17^,^18^,^19^,^20^.

In an earlier study, we compared the amounts of PCBs produced during 13 thermal processes, and found that secondary copper (SeCu) smelting was one of the most important sources of PCBs in China^21^. The Chinese copper industry has grown significantly over the last few decades, driven by increasing domestic copper demand^22^. Recycling and reuse of copper scrap is an important economic activity that offers significant environmental benefits^22^. The contribution of SeCu production to the total production of refined copper increased from 20% in 1975 to 38% in 2010, which indicates that the size of the SeCu industry has grown rapidly^22^. Because of the size of the SeCu industry, and the fact that the processes involved are important sources of PCBs, an understanding of the amounts and mechanisms of PCB formation in SeCu processes could be used to control and decrease PCB emissions. A detailed study of the mechanisms of PCB formation during SeCu smelting could provide this information.

The aim of this study was to gain insight into the potential for fly ash-mediated formation of PCBs in the cooling zone of a SeCu smelter, and the mechanisms involved. Fly ash from a SeCu smelter was used as a reaction matrix in a laboratory-scale system to simulate the thermochemical reactions that produce PCBs under controlled and realistic conditions. The effects of the thermochemical conditions on the formation of PCBs were investigated. The kinetics involved in the formation of the trichlorinated biphenyl (triCB) to decachlorinated biphenyl (decaCB) and total PCBs (ΣPCBs) were explored in detail. Formation mechanisms were explored by characterization of homologue profiles and relevance analyses. Achieving synergistic emission reductions of multiple unintentional POPs is the optimal strategy for sustainable industrial development. Correlation analysis of multiple POPs with similar structures and physicochemical properties could allow for synergistic control of multiple POPs. To evaluate this, PCBs were correlated with other POPs typically formed during SeCu smelting, including polychlorinated dibenzo-*p*-dioxins (PCDDs), polychlorinated dibenzofurans (PCDFs) and polychlorinated naphthalenes (PCNs). The results of this study could be used to develop control methods for emissions of PCBs and other POPs formed during metallurgical processes.

## Results

### Determining the potential for PCB to be formed using simulation experiments

**Figure 1:**
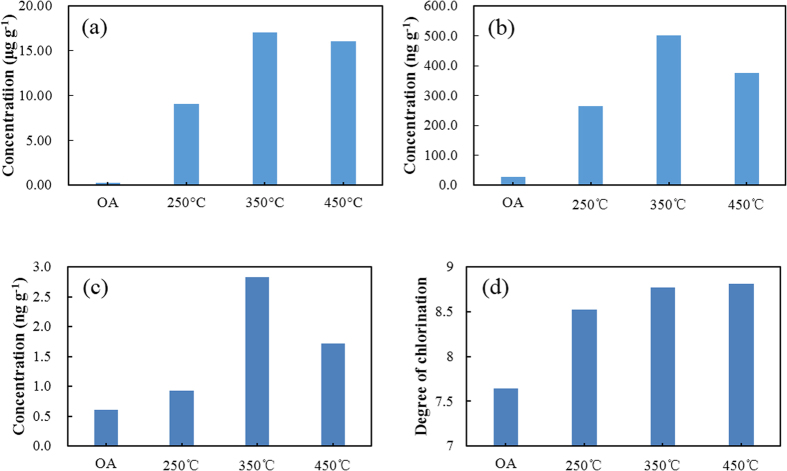
Total (a) polychlorinated biphenyl (PCB) concentrations, (b) dioxin-like PCB concentrations, (c) PCB toxic equivalent concentrations, and (d) degrees of chlorination of the PCBs formed after thermal treatment at 250–450 °C. OA is the original fly ash.

**Figure 2:**
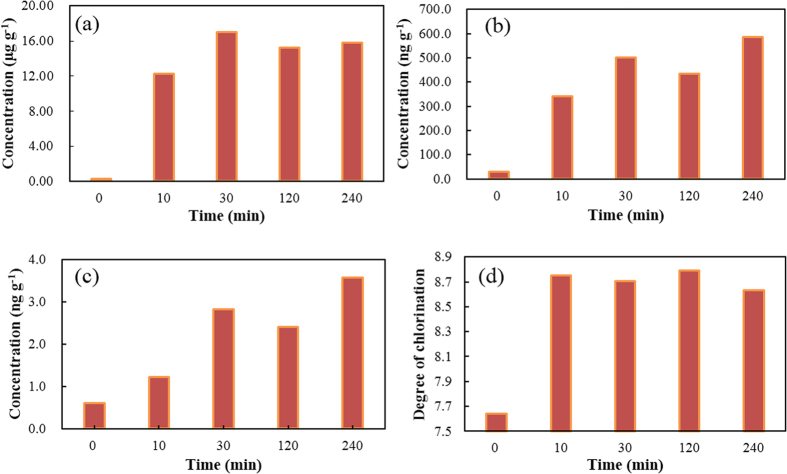
Total (a) PCB concentrations, (b) dioxin-like PCB concentrations, (c) toxic equivalent concentrations, and (d) degrees of chlorination of the PCBs formed after thermal treatment at 350 °C for up to 240 min.

Most PCBs that are produced in industrial thermal process are formed when flue gases cool after they have left the zone where the thermal process takes place. In the present study, the laboratory-scale simulated PCB formation process was designed to mimic the thermal conditions that occur in the cooling zone at an industrial plant. The PCB concentrations in the products were calculated based on the initial weight of raw fly ash (0.2 g) used in the experiments. The PCB concentrations found in the vapour phase samples collected during the thermal reaction tests are shown in Figs 1 and 2. Large quantities of total (Σ) tri- to decaCB (9.0–17 μg g^−1^) were produced during the thermal treatment tests. Approximately 37–69 times more ΣPCBs were formed during the tests than the raw fly ash contained (0.25 μg g^−1^). The dioxin-like PCBs (dl-PCBs) are 12 congeners, for which the World Health Organization has assigned toxicity equivalency factors because they exhibit dioxin-like toxicity^23^. These 12 congeners form a large proportion of the total toxic equivalents (TEQ) in many environmental media, and present considerable health risks. In this study, the Σdl-PCB concentrations were 264–587 ng g^−1^, and 9.4–21 times than that in the raw fly ash (28 ng g^−1^). The TEQs were between 0.93 and 3.6 ng TEQ g^−1^, and 1.5–5.9 times that than in the raw fly ash (0.61 ng TEQ g^−1^).

**Figure 3:**
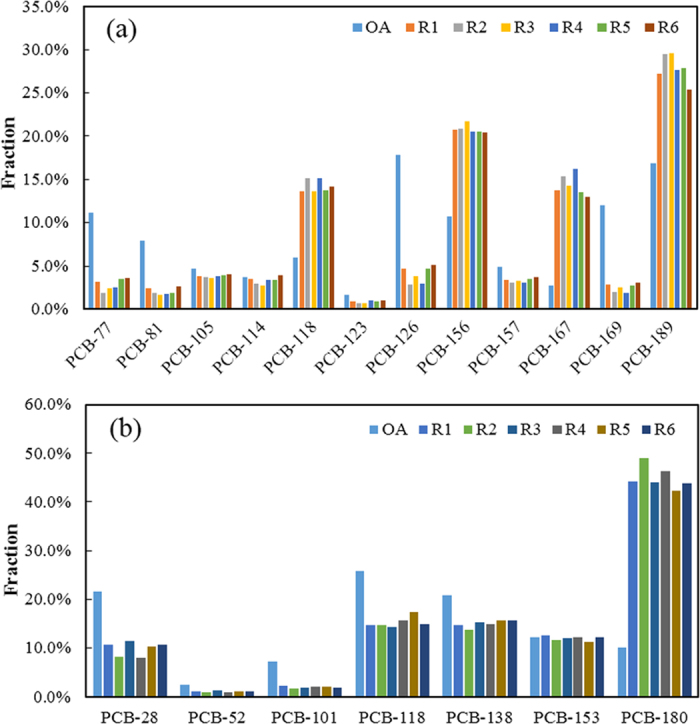
Dioxin-like PCB (dl-PCB) congener profile (a) and indicator PCB congener profile (b) for the products of the simulated SeCu process. The congeners are numbered using the IUPAC-approved system. OA = original fly ash; R1 = 350 °C, 30 min; R2 = 250 °C, 30 min; R3 = 450 °C, 30 min; R4 = 350 °C, 10 min; R5 = 350 °C, 120 min; R6 = 350 °C, 240 min.

The relative contributions of the 12 dl-PCB congeners to the Σdl-PCB concentrations in the thermal treatment products and the raw ash are shown in Fig. 3a. The PCB congeners were numbered using the IUPAC-approved system. The major dl-PCB congeners in the raw ash were PCBs 126 and 169, which comprised 17.8% and 16.8%, respectively, of the Σdl-PCB concentration. As can be seen in Fig. 3a, the dominant dl-PCB congeners after the thermal treatment tests were PCBs 189 and 156, which comprised 27.8% and 20.8%, respectively, of the Σdl-PCB concentrations. Because of their high TEFs, PCBs 126 and 169 were the main contributors to the PCB TEQs in the raw ash and in the products of the thermal treatment tests. The concentrations of the indicator PCB congeners in the products were also determined. Figure 3b shows the relative contributions of the indicator congeners. The dominant congener in the original fly ash was PCB-118, and that in the products was PCB-180.

**Figure 4:**
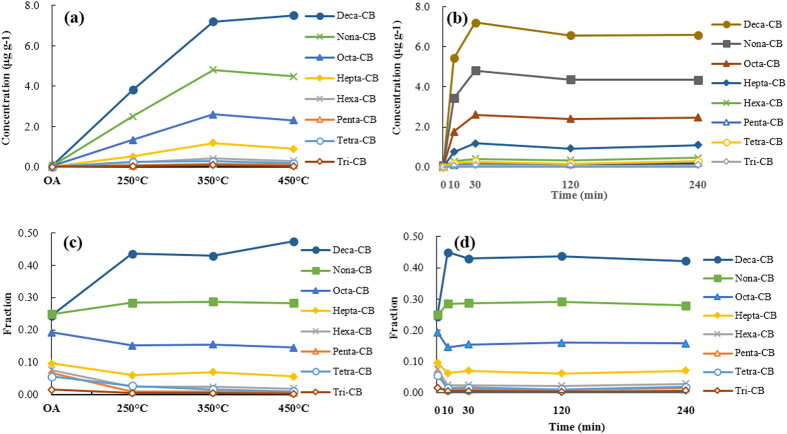
Concentrations of PCB homologues as a function of the (a) reaction temperature and (b) reaction time of the simulated SeCu process. PCB homologue group profiles as a function of the (**c**) reaction temperature and (**d**) reaction time of the simulated SeCu process. CB = chlorinated biphenyl.

The concentrations of the PCB homologues before and after the thermochemical tests are shown in Fig. 4a,b. Tri- to decaCBs were formed during the tests in different quantities. In general, the homologues with more chlorine atoms were formed in higher concentrations. This trend is clearer in Fig. 4c,d, which shows the contributions of the PCB homologue groups to the ΣPCB concentrations. The dominant homologue groups in the raw ash were the nonachlorobiphenyl (nonaCB), decaCB, and octachlorobiphenyl (octaCB), which comprised 25.0, 24.4, and 19.3%, respectively, of the ΣPCB concentration. Large changes were observed in the PCB profile after the thermochemical reaction. DecaCB was the most abundant homologue group after all of the tests, comprising 42.7–47.1% of the ΣPCB concentrations. NonaCB was the second most abundant homologue group after the tests, contributing 28.3–29.1% of the ΣPCB concentrations. The contribution of octaCBs to the ΣPCB concentrations decreased to 15.3–16.1% after the tests, and the contributions of the tri- to heptaCBs to the ΣPCB concentrations were also lower after the tests than in the raw ash.

### Effect of temperature on the formation of PCBs

The relationships between the ΣPCB concentrations, the Σdl-PCB concentrations, the PCB TEQs, and the thermal treatment temperature are shown in Figure 1. There was a large range in the ΣPCB concentrations (9.0–17 μg g^−1^) obtained after thermal treatments at different temperatures (250–450 °C). With a reaction temperature of 350 °C, the ΣPCB concentration was 17 μg g^−1^, which was much higher than that obtained at 250 °C (9.0 μg g^−1^) and slightly higher than that obtained at 450 °C (16 μg g^−1^). For Σdl-PCB, the concentration at a reaction temperature of 350 °C (501.9 ng g^−1^) was much higher than that at 250 °C (264 ng g^−1^) and somewhat lower than that at 450 °C (375 ng g^−1^). When the temperature was increased from 250 to 350 °C, the TEQ also increased (from 0.9 to 2.8 ng TEQ g^−1^). With a further increase in the temperature to 450 °C, the TEQ decreased slightly (to 2.4 ng TEQ g^−1^). The degree of chlorination of the PCBs that were formed at each temperature was also determined. The mean degree of chlorination is defined by Weber *et al.*^24^ as the average number of chlorine atoms in a polychlorinated aromatic molecule (e.g., a PCB molecule) in a sample. In this study, the degree of chlorination increased as the temperature increased (Fig. 1). The PCB profile changed noticeably as the temperature was increased from 250 to 450 °C. The concentrations of most of the homologue groups (tri- to nonaCBs) were highest when the reaction temperature was 350 °C, but the decaCB concentration increased further when the reaction temperature was increased to 450 °C (Fig. 4a).

### Formation of PCBs as a function of the reaction time

The formation of PCBs was studied at reaction times between 10 and 240 min (Fig. 2). Initially, the ΣPCB concentration increased remarkably from 0.25 μg g^−1^ in the raw ash to 12 μg g^−1^ after 10 min of thermal treatment. Then, the ΣPCB concentration increased more slowly, reaching a maximum concentration of 17.03 μg g^−1^ at a reaction time of 30 min. The ΣPCB concentration remained relatively constant when the reaction time was increased beyond 30 min. The trends in the Σdl-PCB and TEQs were slightly different. With a reaction time of 10 min, the Σdl-PCB concentration did not increase as much as the ΣPCB concentration. Instead, the Σdl-PCB concentration reached a maximum at a reaction time of 30 min and then remained relatively constant. The trend in the TEQs was similar to the trend in the Σdl-PCB concentration. For each individual PCB homologue group, the concentrations and contributions to the ΣPCB concentrations after different reaction times are shown in detail in Fig. 4b,d. The maximum concentrations for all of the homologue groups (tri- to decaCB) were reached within a reaction time of 30 min, and then the concentrations remained constant as the reaction time increased.

## Discussion

It is widely recognised that heterogeneous catalysis reactions occur on fly ash in the cooling zone of combustors and smelters^25^. Consequently, in a number of studies, fly ash has been used in thermal experiments as a matrix that was as close to a “real” active surface in thermal processes^25^,^26^,^27^. The formation of PCBs and other chlorinated organic chemicals is likely favoured by the presence of carbon, chlorine, and transition metals such as Cu on the surface of fly ash^26^,^27^,^28^,^29^. High concentrations of carbon, chlorine, oxygen, and transition metals especially for Cu on the prepared fly ash surface have been determined in the published work^30^,^31^. The surface properties of the fly ash used in this study were determined in the published work^30^,^31^ and the fly ash was found to have a high surface area that would allow relatively large quantities of POPs to be adsorbed. The cooling zone of an incinerator or a smelter comes before the filter bag or other air pollution control devices, and is the main formation zone of dioxins and dioxin-like compounds because the temperature is favourable for formation of these compounds. Additionally, the flue gas containing fly ash particles enters the cooling zone where gas-particle reactions of PCBs might occur. Fly ash remains in the flue gas until it is removed by air pollution control devices. We speculated that fly ash could contribute to PCBs formation in the cooling zone of a SeCu smelter. The results of our simulation experiments supported the formation of PCBs from fly ash in SeCu processes.

In China, fly ash is often recycled to recover residual metal. In consideration of the contribution that fly ash makes to PCB TEQs, the recycling of fly ash should be reconsidered because of the potential for environmental pollution. Some studies have found that thermal treatment of fly ash under an inert atmosphere achieves high removal of PCB residues^24^,^32^,^33^,^34^. However, the results of the present study suggested that exposure of fly ash to air for several minutes promoted the formation of PCBs. Therefore, thermal treatment of fly ash without controlling the treatment atmosphere could increase the risk of formation of dl-PCBs rather than remove PCB residues. Therefore, control of the treatment atmosphere is recommended in thermal treatment of fly ash.

An understanding of the mechanisms for unintentional formation of PCBs could be used to guide development of methods for control and reduction of PCB levels in the environment. However, the formation mechanisms of PCBs in industrial processes are not well understood. The results of the present study suggest fly ash mediated a chlorination mechanism for PCB formation. Compared with the homologue distribution before thermal treatment, PCB homologue groups with more chlorine atoms contributed more to the ΣPCB concentration and those with fewer chlorine atoms contributed less to the ΣPCB concentration. This suggests that a chlorination reaction, where lower chlorinated congeners were converted into higher chlorinated congeners, was involved in the formation of PCBs during thermal treatment. The mechanistic relationships between the PCB homologue groups were investigated by determining the Pearson correlation coefficients (*R*) for the relationships between the concentrations of the different PCB homologue groups. The concentrations of adjacent PCB homologue groups correlated well (Table 1). These results support the conclusion that a chlorination pathway plays a part in the formation of PCBs during thermal processes. Some previous studies have also found that *de novo* synthesis, including the oxychlorination and breakdown of carbon, is a large contributor to PCB formation during industrial thermal processes^27^,^29^,^35^. Therefore, the large quantities of PCB formed in thermal reactions might be formed through multiple pathways including chlorination and *de novo* synthesis.

Key parameters that influence PCB emission, profiles, and formation in SeCu process have not yet been identified. In the present study, simulation experiments were performed to determine the effect of the temperature on the concentrations of ΣPCB and PCB homologue groups formed during thermal treatment. Among the temperatures that were tested, 350 °C most favoured the formation of the PCBs. The ΣPCB concentration increased greatly when the reaction temperature was increased from 250 to 350 °C, and decreased slightly when the temperature was increased further to 450 °C. Similar trends were also seen for dl-PCB and TEQs as the temperature was increased.

Moreover, the results indicated that thermal treatment of fly ash at higher temperatures promoted PCB chlorination. The degree of chlorination of PCBs formed at different temperatures was also studied. The degree of chlorination increased as the treatment temperature increased (Fig. 1). The contributions of the tri- to decaCB homologue groups to the ΣPCB concentrations were very different at different temperatures. Higher temperatures (450 °C and possibly even higher) were found to favour the production of higher chlorinated PCBs, whereas the production of lower chlorinated PCBs was favoured at 350 °C. We presume that different temperatures favour the production of different PCB homologue groups because their reactions have different rate constants at different temperatures.

In our tests we found that large quantities of PCBs were formed within the first 30 min, and then the production level remained constant with further increases in the reaction time. Thermochemical reactions on fly ash that form or/and degrade PCBs are highly dependent on treatment parameters^27^,^28^,^32^,^33^. The observed phenomena may result from simultaneous formation and degradation processes occurring on the fly ash. That is, all of the PCB homologue groups may form at the beginning of the thermal treatment, and equilibrium between formation and degradation is reached within 30 min.

It is widely recognized that fingerprint analysis is an excellent tool for linking sources with their potential impact on the environment. Therefore, we compared the congener patterns of the produced PCBs with patterns in other matrices. The congener pattern was very different from those in PCB technical mixtures^36^. The dominant indicator congeners were PCB-28 and PCB-138 in the technical mixtures, while the dominant congener produced after thermal treatment was PCB-180, which has a higher degree of chlorination than PCB-28 and PCB-138. The homologue profiles of the PCBs formed after thermal treatment and those in technical mixtures were compared. Takasuga *et al.*^37^ determined the homologue profiles for PCB technical mixtures from Japan (Kanechlor), Germany (Clophen), the USA (Aroclor), Russia (Sovol) and Poland (Chlorofen), and found they were similar. The dominant homologue in each technical mixture varied with the mass fraction of chlorine in the mixture. The PCB homologue profiles found in the present study (see Supplementary Fig. S1 online) differed from those of the PCB technical mixtures. This difference could be caused by the dominance of the highly chlorinated homologue (decaCB) in the PCBs formed after thermal treatment. Additionally, the distribution of homologue decreased as the number of chlorine atoms in each homologue group decreased. These patterns could be useful for identifying sources of PCB contamination in environmental matrices.

**Figure 5:**
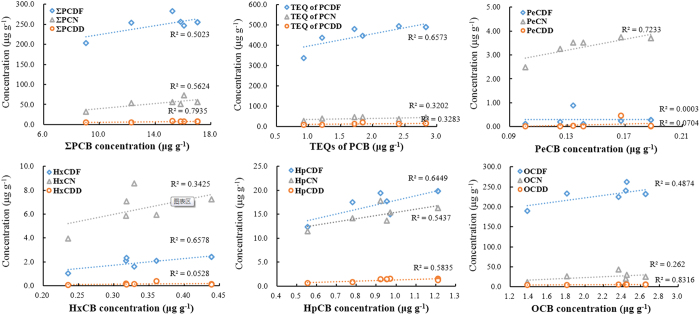
Chlorinated dibenzo-*p*-dioxin (CDD), chlorinated dibenzofuran (CDF), and chlorinated naphthalene (CN) homologue concentrations (data of PCDD/Fs and PCNs have been reported in previous work^30^,^31^) plotted against the corresponding chlorinated biphenyl (CB) homologue concentrations. TEQ = toxic equivalent. The prefixes are Σ = total, P = poly, Pe = penta, Hx = hexa, Hp = hepta, and O = octa.

A number of unintentionally produced POPs can be formed and released during industrial processes. For development of sustainable industrial process, emissions of multiple unintentionally produced POPs should be decreased simultaneously. In addition to PCBs, unintentionally produced POPs include PCDDs, PCDFs and PCNs, which have similar structures and physicochemical properties to PCBs. We have investigated the production of PCDDs, PCDFs, and PCNs using simulations that are described in previous studies^30^,^31^. Correlation analyses were performed between the total concentrations and contributions of the different homologue groups of these POPs, and used to attempt to identify mechanisms of formation for PCBs and other POPs. This information could help decrease simultaneous emissions of multiple POPs from SeCu smelters. Significant correlations were found between the PCB concentrations and TEQs, and the PCDD, PCDF, and PCN concentrations and TEQs. As shown in Fig. 5, the *R*^*2*^values for the correlations between the PCB concentration and the PCDD, PCDF, and PCN concentrations were 0.79, 0.50, and 0.56, respectively. These results show that the PCB concentrations correlate better with the PCDD concentrations than with the PCN and PCDF concentrations. The correlations between the PCB homologue group concentrations and the corresponding homologue groups of other POPs were also investigated in detail to further elucidate the mechanistic relationships between the formation mechanisms of PCBs and other POPs. For example, strong correlations were found between the penta- to octachlorinated homologue concentrations of the PCBs and the other POPs (Fig. 5). This information will help with decreasing emissions of multiple POPs from thermal-related processes. Among all the pollutants investigated, the PCDD/Fs made a large contribution to the total TEQ. The TEQ contribution from PCDFs was 178–372 times higher that of PCBs. These results agree with those from earlier studies on waste incineration processes, which showed low contributions from PCBs compared to PCDDs/Fs^38^,^39^. Remarkable differences were observed in comparison with both environmental and biological samples, such as soils, sediments, human milk and fish, because PCBs in these matrices may contribute up to 77% of the total TEQ^37^,^40^,^41^. These results may be useful for PCB environmental contamination source apportioning.

In conclusion, the formation of PCBs, and especially of higher chlorinated homologues, was strongly promoted in the simulated thermochemical reactions. Correlation analysis and comparison of homologue and congener patterns before and after thermochemical reactions suggested lower chlorinated homologues were converted to higher chlorinated homologues. The chlorination reaction was favoured at higher temperatures in the investigated temperature range. Moreover, the formation of PCBs was closely related to the formation of other POPs, including PCDDs, PCDFs, and PCNs, indicating there are close relationships between the formation mechanisms for different types of POPs. The results of this study improve the understanding of the mechanisms involved in the formation of PCBs in thermal industrial processes, and could be applied to developing methods for simultaneous control of emissions of multiple POPs from thermal industrial plants.

## Methods

### Experimental design

Fly ash collected from a reverberatory furnace in a SeCu smelter in Eastern China was used as the matrix in the thermal experiments. The production capacity of the furnace was 110 tonnes (110 000 kg). The surface composition and properties of the ash were determined in earlier work^30^,^31^.

Simulation experiments were performed as described previously^30^,^31^. Briefly, the simulation system was a quartz tube reactor (length 60 cm, Ø 4.5 cm) within a tube furnace, which provided even heating and precise temperature control. To simulate ash particles in the cooling zone of a SeCu smelter, the fly ash (0.2 g) was placed in a fixed bed on a porcelain boat in the reactor. A constant stream of air flowed over the ash to mimic the atmosphere in the cooling zone. Experiments were performed at 250–450 °C to encompass the temperatures at which PCBs can form in the cooling zone of SeCu smelting. Fly ash particles can have residence times of hours in the cooling zone of a SeCu plant^26^, but it has not yet been determined whether PCBs can be formed at shorter reaction times. Therefore, reaction times ranging from several minutes to several hours (10, 30, 120, and 240 min) were used. The results were used to study the kinetics of PCB formation and the relationship between the amount of PCBs formed and the residence time. The outlet gas was quenched as soon as it left the reactor by passing it through three absorption bottles, each of which contained 50 mL of toluene and were cooled on ice. The reaction conditions are presented in published work^30^,^31^.

### Analytical methods

The toluene from the traps and 0.2 g of the solid residue were analysed for PCBs by isotope dilution high-resolution gas chromatography and high-resolution mass spectrometry (HRGC/HRMS). Each toluene trap sample or residue sample was spiked with a mixture of ^13^C_12_-labelled PCB internal standards (1668A-LCS; Cambridge Isotope Laboratories, Andover, MA, USA). Before extraction, each solid sample was digested in hydrochloric acid and then freeze-dried. Each residue sample was Soxhlet extracted with 250 mL of toluene for 24 h. Each extract (or toluene trap sample) was cleaned by passing it through a series of chromatographic columns, including an acidified silica gel column, a multilayer silica gel column, and a basic alumina column. The cleaned extract was then concentrated, first by rotatory evaporation and then under a gentle stream of nitrogen, to approximately 20 μL. The initial PCB concentration in the raw fly ash was determined so that the amount of PCBs formed in each test could be calculated in comparison. The raw fly ash was analysed using the same method as for the solid residue samples. Each cleaned sample extract was spiked with a mixture of ^13^C_12_-labelled PCB recovery standards (1668A-IS; Cambridge Isotope Laboratories) before analysis by HRGC/HRMS. The HRGC/HRMS analyses were carried out using an Agilent 6890 gas chromatograph (Agilent Technologies, Santa Clara, CA, USA) coupled to an AutoSpec Ultima mass spectrometer (Waters Micromass, Milford, MA, USA). The PCB congeners were separated using a DB-5 capillary column (60 m × 0.25 mm i.d., 0.25-μm film thickness; Agilent Technologies). The HRMS was used in selected ion monitoring mode with a resolution of >10000. Electron ionization was performed with an electron energy of 35 eV. The source temperature was 250 °C.

### Quality control and assurance

A blank test was performed after every three sample tests, and used to determine the quantity of PCBs produced when no reactants were present. The reactor was kept at 350 °C for 10 min in each blank test, and the gas that passed through the system was collected in the same way as for a sample. The PCB concentrations in the toluene traps from all of the blank tests were less than 0.9% of the concentrations found in the toluene traps from the sample tests. Therefore, no blank correction was required. The PCBs found in the vapour phase samples contributed between 87.88 and 99.98% of the ΣPCB concentrations in the samples. Therefore, in the PCB formation experiments, the PCB concentrations in the vapour phase samples were used to represent the ΣPCB concentrations. Experiment 1, in which the middle reaction temperature and time were used, was performed in triplicate. The relative standard deviation of the PCB concentrations in triplicate analyses of sample media was less than 5%. Recoveries for spiked internal standards were between 34 and 160%, proving that the sample treatment methods were satisfactory according to the US EPA 1668 method^42^.

## Additional Information

**How to cite this article**: Jiang, X. *et al.* Formation of Polychlorinated Biphenyls on Secondary Copper Production Fly Ash: Mechanistic Aspects and Correlation to Other Persistent Organic Pollutants. *Sci. Rep.* **5**, 13903; doi: 10.1038/srep13903 (2015).

## Supplementary Material

Supplementary Materials

## Figures and Tables

**Table 1:** Pearson correlation coefficients for the relationships between the polychlorinated biphenyl homologue groups.

	MonoCB	DiCB	TriCB	TetraCB	PentaCB	HexaCB	HeptaCB	OctaCB	NonaCB	DecaCB
MonoCB	1									
DiCB	0.979^**^	1								
TriCB	–0.155	0.021	1							
TetraCB	–0.276	–0.120	0.798^*^	1						
PentaCB	0.141	0.259	0.884^**^	0.608	1					
HexaCB	–0.078	0.038	0.907^**^	0.612	0.969^**^	1				
HeptaCB	–0.320	–0.240	0.795^*^	0.504	0.847^*^	0.947^**^	1			
OctaCB	–0.237	–0.211	0.608	0.277	0.772^*^	0.866^*^	0.958^**^	1		
NonaCB	–0.313	–0.299	0.540	0.194	0.682	0.806^*^	0.935^**^	0.988^**^	1	
DecaCB	–0.287	–0.303	0.395	0.059	0.558	0.696	0.853^*^	0.937^**^	0.974^**^	1

CB = chlorinated biphenyl

## References

[b1] Holmes D. C., Simmons J. H. & Tatton J. O. Chlorinated hydrocarbons in British wildlife. Nature 216, 227–229 (1967). 6066133 10.1038/216227a0

[b2] Ratcliff., Da. Decrease in eggshell weight in certain birds of prey. Nature 215, 208–210 (1967). 6049131 10.1038/215208a0

[b3] Arisawa K. *et al.* Dietary intake of PCDDs/PCDFs and coplanar PCBs among the Japanese population estimated by duplicate portion analysis: A low proportion of adults exceed the tolerable daily intake. Environ. Res. 108, 252–259 (2008). 18692182 10.1016/j.envres.2008.06.011

[b4] Koopman-Esseboom C. *et al.* Effects of dioxins and polychlorinated biphenyls on thyroid hormone status of pregnant women and their infants. Pediatr. Res. 36, 468–473 (1994). 7816522 10.1203/00006450-199410000-00009

[b5] Osius N., Karmaus W., Kruse H. & Witten J. Exposure to polychlorinated biphenyls and levels of thyroid hormones in children. Environ. Health Persp. 107, 843–849 (1999). 10.1289/ehp.99107843 PMC1566609 10504153

[b6] Safe S. Toxicology, structure-function relationship, and human and environmental-health impacts of polychlorinated-biphenyls - progress and problems. Environ. Health Perspect. 100, 259–268 (1993). 8354174 10.1289/ehp.93100259 PMC1519588

[b7] Schantz S. L., Widholm J. J. & Rice D. C. Effects of PCB exposure on neuropsychological function in children. Chem. Eng. J. 111, 357–376 (2003). 10.1289/ehp.5461 PMC1241394 12611666

[b8] Conference of the Parties to the Stockholm Convention. Preliminary assessment of efforts made toward the elimination of polychlorinated biphenyls Report No. UNEP/POPS/COP.7/INF/9, 1-40 (UNEP, Geneva, Switzerland 2015).

[b9] Praipipat P. Source apportionment of polychlorinated biphenyls in New Jersey air and Delaware River sediments. Rutgers University-Graduate School-New Brunswick (2014).

[b10] Praipipat P., Rodenburg L. A. & Cavallo G. J. Source apportionment of polychlorinated biphenyls in the sediments of the Delaware River. Environ. Sci. Technol. 47, 4277–4283 (2013). 23586856 10.1021/es400375e

[b11] Hu D. & Hornbuckle K. C. Inadvertent Polychlorinated Biphenyls in Commercial Paint Pigments†. Environ. Sci. Technol. 44, 2822–2827 (2009). 19957996 10.1021/es902413k PMC2853905

[b12] Anezaki K., Kannan N. & Nakano T. Polychlorinated biphenyl contamination of paints containing polycyclic-and Naphthol AS-type pigments. Environ. Sci. Pollut. Res. 10.1007/s11356-014-2985-6 (2014). 24809497

[b13] Huang J. *et al.* Unintentional formed PCDDs, PCDFs, and DL-PCBs as impurities in Chinese pentachloronitrobenzene products. Environ. Sci. Pollut. Res. 10.1007/s11356-014-3507-2 (2014). 25167828

[b14] Liu W., Tao F., Zhang W., Li S. & Zheng M. Contamination and emission factors of PCDD/Fs, unintentional PCBs, HxCBz, PeCBz and polychlorophenols in chloranil in China. Chemosphere 86, 248–251 (2012). 22018590 10.1016/j.chemosphere.2011.09.034

[b15] Liu W. *et al.* Formation and contamination of PCDD/Fs, PCBs, PeCBz, HxCBz and polychlorophenols in the production of 2,4-D products. Chemosphere 92, 304–308 (2013). 23601123 10.1016/j.chemosphere.2013.03.031

[b16] Dyke P. H., Foan C. & Fiedler H. PCB and PAH releases from power stations and waste incineration processes in the UK. Chemosphere 50, 469–480 (2003). 12685746 10.1016/s0045-6535(02)00627-6

[b17] Sakai S. I., Hayakawa K., Takatsuki H. & Kawakami I. Dioxin-like PCBs released from waste incineration and their deposition flux. Environ. Sci. Technol. 35, 3601–3607 (2001). 11783634 10.1021/es001945j

[b18] Ba T. *et al.* Estimation and characterization of PCDD/Fs and dioxin-like PCBs from secondary copper and aluminum metallurgies in China. Chemosphere 75, 1173–1178 (2009). 19329140 10.1016/j.chemosphere.2009.02.052

[b19] Nie Z. *et al.* A preliminary investigation of unintentional POP emissions from thermal wire reclamation at industrial scrap metal recycling parks in China. J. Hazard. Mater. 215, 259–265 (2012). 22436338 10.1016/j.jhazmat.2012.02.062

[b20] Nie Z., Liu G., Liu W., Zhang B. & Zheng M. Characterization and quantification of unintentional POP emissions from primary and secondary copper metallurgical processes in China. Atmos. Environ. 57, 109–115 (2012).

[b21] Liu G. *et al.* Atmospheric emission of polychlorinated biphenyls from multiple industrial thermal processes. Chemosphere 90, 2453–2460 (2013). 23246728 10.1016/j.chemosphere.2012.11.008

[b22] Zhang L., Yang J., Cai Z. & Yuan Z. Analysis of copper flows in China from 1975 to 2010. Sci. Total Environ. 478, 80–89 (2014). 24530587 10.1016/j.scitotenv.2014.01.070

[b23] Van den Berg M. *et al.* The 2005 World Health Organization reevaluation of human and mammalian toxic equivalency factors for dioxins and dioxin-like compounds. Toxicol. Sci. 93, 223–241 (2006). 16829543 10.1093/toxsci/kfl055 PMC2290740

[b24] Weber R. *et al.* Dechlorination and destruction of PCDD, PCDF and PCB on selected fly ash from municipal waste incineration. Chemosphere 46, 1255–1262 (2002). 12002448 10.1016/s0045-6535(01)00268-5

[b25] Milligan M. S. & Altwicker E. The relationship between *de novo* synthesis of polychlorinated dibenzo-p-dioxins and dibenzofurans and low-temperature carbon gasification in fly ash. Environ. Sci. Technol. 27, 1595–1601 (1993).

[b26] Addink R. & Olie K. Mechanisms of formation and destruction of polychlorinated dibenzo-*p*-dioxins and dibenzofurans in heterogeneous systems. Environ. Sci. Technol. 29, 1425–1435 (1995). 22276861 10.1021/es00006a002

[b27] Pekárek V. *et al.* Matrix effects on the *de novo* synthesis of polychlorinated dibenzo-p-dioxins, dibenzofurans, biphenyls and benzenes. Chemosphere 68, 51–61 (2007). 17291561 10.1016/j.chemosphere.2006.12.069

[b28] Pekárek V., Grabic R., Marklund S., Punčochář M. & Ullrich J. Effects of oxygen on formation of PCB and PCDD/F on extracted fly ash in the presence of carbon and cupric salt. Chemosphere 43, 777–782 (2001). 11372865 10.1016/s0045-6535(00)00433-1

[b29] Weber R. *et al.* Formation of PCDF, PCDD, PCB, and PCN in *de novo* synthesis from PAH: Mechanistic aspects and correlation to fluidized bed incinerators. Chemosphere 44, 1429–1438 (2001). 11513122 10.1016/s0045-6535(00)00508-7

[b30] Jiang X., Liu G., Wang M. & Zheng M. Fly ash-mediated formation of polychlorinated naphthalenes during secondary copper smelting and mechanistic aspects. Chemosphere 119, 1091–1098 (2015). 25460747 10.1016/j.chemosphere.2014.09.052

[b31] Wang M., Liu G., Jiang X., Xiao K. & Zheng M. Formation and potential mechanisms of polychlorinated dibenzo-p-dioxins and dibenzofurans on fly ash from a secondary copper smelting process. Environ. Sci. Pollut. Res. 22, 8747–8755 (2015). 10.1007/s11356-014-4046-6 25572269

[b32] Lundin L. & Marklund S. Thermal degradation of PCDD/F, PCB and HCB in municipal solid waste ash. Chemosphere 67, 474–481 (2007). 17109915 10.1016/j.chemosphere.2006.09.057

[b33] Stach J., Pekárek V. r., Grabic R., Lojkásek M. & Pacáková V. Dechlorination of polychlorinated biphenyls, dibenzo-p-dioxins and dibenzofurans on fly ash. Chemosphere 41, 1881–1887 (2000). 11061310 10.1016/s0045-6535(00)00144-2

[b34] Hung P. C., Chen Q. H. & Chang M. B. Pyrolysis of MWI fly ash - Effect on dioxin-like congeners. Chemosphere 92, 857–863 (2013). 23714152 10.1016/j.chemosphere.2013.04.042

[b35] Katami T., Yasuhara A., Okuda T. & Shibamoto T. Formation of PCDDs, PCDFs, and coplanar PCBs from polyvinyl chloride during combustion in an incinerator. Environ. Sci. Technol. 36, 1320–1324 (2002). 11944687 10.1021/es0109904

[b36] Breivik K., Sweetman A., Pacyna J. M. & Jones K. C. Towards a global historical emission inventory for selected PCB congeners — a mass balance approach: 1. Global production and consumption. Sci. Total Environ. 290, 181–198 (2002). 12083709 10.1016/s0048-9697(01)01075-0

[b37] van Bavel B. *et al.* Levels of PCBs in the aquatic environment of the Gulf of Bothnia: benthic species and sediments. Mar. Pollut. Bull. 32, 210–218 (1996).

[b38] Abad E., Martínez K., Caixach J. & Rivera J. Polychlorinated dibenzo-p-dioxins, dibenzofurans and ‘dioxin-like’ PCBs in flue gas emissions from municipal waste management plants. Chemosphere 63, 570–580 (2006). 16216299 10.1016/j.chemosphere.2005.08.026

[b39] Sakurai T., Weber R., Ueno S., Nishino J. & Tanaka M. Relevance of coplanar PCBs for TEQ emission of fluidized bed incineration and impact of emission control devices. Chemosphere 53, 619–625 (2003). 12962711 10.1016/S0045-6535(03)00536-8

[b40] Strandberg B. *et al.* Occurrence, sedimentation, and spatial variations of organochlorine contaminants in settling particulate matter and sediments in the northern part of the Baltic Sea. Environ. Sci. Technol. 32, 1754–1759 (1998).

[b41] Becher G., Haug L. S. & Thomsen C. World-wide comparison on the quality of analytical determinations of PCDDs/PCDFs and dioxin-like PCBs in food. Talanta 63, 1115–1122 (2004). 18969541 10.1016/j.talanta.2004.05.038

[b42] U.S EPA. Method 1668B Chlorinated Biphenyl Congeners in Water, Soil, Sediment, Biosolids, and Tissue by HRGC/HRMS. U.S. Environmental Protection Agency, Washington, DC, EPA/1821/R-1608/1020 (2008).

