# Supplementary material for: Formation of Polychlorinated Biphenyls on Secondary Copper Production Fly Ash: Mechanistic Aspects and Correlation to Other Persistent Organic Pollutants
Source: Sci Rep. 2015 Sep 16;5:13903. doi: 10.1038/srep13903 (PMC4570990; doi:10.1038/srep13903)
Supplement: Supplementary Materials [file srep13903-s1.doc]

**Supplementary material**

Formation of Polychlorinated Biphenyls on Secondary Copper Production Fly Ash: Mechanistic Aspects and Correlation to Other Persistent Organic Pollutants

Xiaoxu Jiang, Guorui Liu^*^, Mei Wang, Minghui Zheng

State Key Laboratory of Environmental Chemistry and Ecotoxicology, Research Center for Eco-Environmental Sciences, Chinese Academy of Sciences, P.O. Box 2871, Beijing 100085, China

^*^ Corresponding authors. Tel: +86 10 6284 9356; fax: +86 10 6284 9172

E-mail address: [grliu@rcees.ac.cn](mailto:grliu@rcees.ac.cn)

**Contents:**

Figure S1. PCB homologue profiles in the products of the simulated process. R1=350°C, 30 min; R2=250°C, 30 min; R3=450°C, 30 min; R4=350°C, 10 min; R5=350°C, 120 min; R6=350°C, 240 min; CB = chlorinated biphenyl.


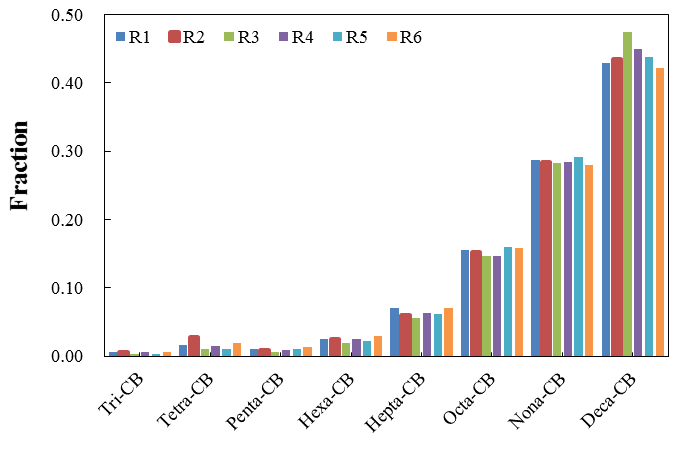


Figure S1. PCB homologue profiles in the products of the simulated process. R1=350°C, 30 min; R2=250°C, 30 min; R3=450°C, 30 min; R4=350°C, 10 min; R5=350°C, 120 min; R6=350°C, 240 min; CB = chlorinated biphenyl.
